# Blockchain integration for in-vehicle CAN bus intrusion detection systems with ISO/SAE 21434 compliant reporting

**DOI:** 10.1038/s41598-024-58694-4

**Published:** 2024-04-08

**Authors:** Tudor Andreica, Adrian Musuroi, Alfred Anistoroaei, Camil Jichici, Bogdan Groza

**Affiliations:** Faculty of Automation and Computers, Politehnica University of Timisoara, 300223 Timisoara, Romania

**Keywords:** Software, Computer science, Information technology

## Abstract

The development of Intrusion Detection Systems (IDS) for in-vehicle buses has gained a lot of momentum in recent years as the number of reported vulnerabilities and the degree of interconnectivity for modern vehicles are on the rise. Since intrusion detection is resource consuming, it can be performed on computationally capable Android head units that are now present inside vehicles. Moreover, these units are connected to the internet, which enables the use of more complex algorithms that run in cloud environments. In this work we develop one such approach: an IDS that consists of a locally installed copy, running on head units, and a centralized instance of it that runs in the cloud and monitors traffic for groups of similar vehicles. Additionally, the centralized instance is part of a cloud service for intrusion detection which is continuously updated with the most recent types of attacks. The classification results of the cloud-based service are further analyzed by an incident response team which confirms the presence of known attacks, analyzes new types of attacks and assesses their impact. The output of this activity is stored on the Blockchain as ISO/SAE 21434 compliant reports, ensuring the transparency and traceability of the reported incidents.

## Introduction

The automotive industry is evolving day by day and is changing in so many ways. In the last decade, the number of ECUs (Electronic Control Units) increased to more than one hundred in high-end vehicles due to the introduction of new technologies and functionalities. New automotive trends, such as electrification or automated driving, will increase the use of software and controllers even more. The communication between ECUs, which is currently much more limited to in-vehicle networks, will be extended to the outer world as vehicle-to-vehicle, vehicle-to-infrastructure or vehicle-to-cloud communications become more prevalent. All these recent technologies will require proper security mechanisms to protect drivers, passengers and other road users against security attacks that can lead to undesired events.

The automotive industry is already focusing on cybersecurity aspects, which have to be part of the entire life-cycle of modern vehicles, from development to decommissioning. The ISO/SAE 21434^1^ standard was released in 2021 and describes the cybersecurity process that has to be established in the automotive industry. Much earlier, the AUTomotive Open System ARchitecture (AUTOSAR) community also released a set of specifications that address security aspects for secure onboard communication^2^, introducing mechanisms for authentication and freshness of CAN (Controller Area Network) messages, while specifications for an Intrusion Detection System Manager (IdsM) on in-vehicle ECUs were later added^3^. Such systems will be used in all future vehicles to detect attacks on various communication layers ranging from in-vehicle buses, such as the CAN bus, to wireless interfaces like WiFi or 5G. Needless to say, once detected, such attacks need to be reported and the corresponding information securely stored. Blockchain is a modern technology that offers several key advantages for this purpose, among which the following three seem to be the most important: immutability, decentralized storage and the traceability of reporting actors. Immutability, assured by the one-way nature of cryptographic hash functions and by the fact that each block added to the chain contains the hash of the previous block, makes the Blockchain resilient against data alteration attacks. Since the Blockchain can be stored in a distributed manner, it makes the whole reporting process more transparent, as multiple actors can be involved in verifying the integrity of the reported data. Indeed, it is expected that an automotive scenario will involve a large number of vehicles and manufacturer’s operatives that will contribute to the incident reporting process. Traceability allows to verify the actors (autonomous cars or the incident response team) that are responsible for generating the data and the attack reports. Without being able to trace the source of such information, adversaries that report false attack data or potentially corrupted reports will greatly hinder the entire process.

**Figure 1:**
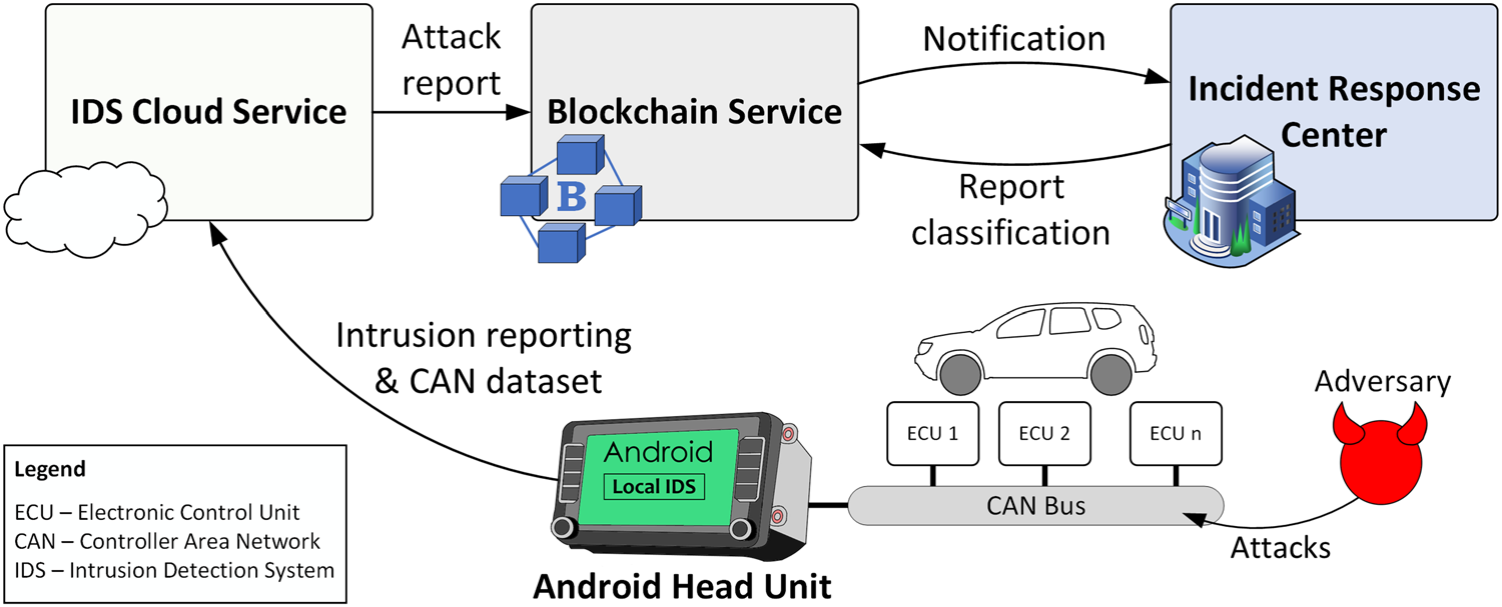
Overview of the proposed intrusion reporting system.

In the light of the recently emerged standards, i.e., ISO 21434^1^ and AUTOSAR IdsM^3^, we try to respond to this novel necessity from the automotive domain by using a highly successful modern technology, i.e., Blockchain. This technology can be used for the secure management of the IDS reports, as it provides a decentralized means of storage. We propose an IDS tailored for attacks on CAN networks, which uses a locally installed instance and a separate cloud-based instance with Blockchain incident reporting capabilities. The local IDS is meant to run on the head unit of each vehicle, while the cloud-based IDS is centralized and operates with data collected from multiple vehicles. The cloud service is updated with the latest discovered attacks and the detection results are stored via the *Blockchain Service* as ISO/SAE 21434 reports, based on the analysis of specialized personnel (since this type of impact assessment can be performed only with the help of human expertise). The incident response team is responsible to verify the presence of attacks, report new types of attacks, update the cloud-based IDS with the new attack types and perform the impact analysis on the newly identified attacks. A high-level representation of the concept is suggested in Fig. 1, showing the four actors in our system assigned with the previously described tasks, i.e., the *Android Head Unit*, the *IDS Cloud Service*, the *Blockchain Service* and the *Incident Response Center*.

The contributions of our work can be summarized as follows: (i) we propose a cloud-based IDS that takes benefit of the computationally capable Android head units inside cars and of the cloud infrastructure, (ii) to illustrate how intrusion detection extends from one vehicle to another, i.e., transfer learning, we use real data collected from 3 identical vehicles that is augmented with adversary traffic and prove that a learned attack from one of the vehicles can be identified on the others, (iii) we illustrate the computational performances of the intrusion detection algorithms by providing benchmarks performed both on the Android units as well as in the cloud, (iv) we organize the attack information and risk assessment into ISO/SAE 21434 compliant reports in order to point out the specific impact, (v) we present a proof of concept application which uses Blockchain as a decentralized mean to report intrusions and the resulting risk-assessment. To sum up the contributions, our work tries to accommodate ISO/SAE 21434 reporting into the Blockchain for a transfer learning IDS that runs both locally on Android head units inside cars as well as in the cloud (allowing specialized staff to analyze incident-related data).

## Related work

Recent attacks have proved serious concerns as vehicles were manipulated remotely by exploiting their web browser as the entry point^4^ and compromising the over-the-air (OTA) software update functionality to attack ECUs^5^. Therefore, the need for cybersecurity measures in modern vehicles became indisputable and generated a lot of interest for the research community. There are multiple attack surfaces that have to be protected in modern vehicles, the in-vehicle networks being the most critical ones and the most commonly exploited^6,7^. Consequently, in recent years, many security solutions for in-vehicle communication have been proposed^8^. Some of the proposed solutions call for cryptography and are based on message authentication^9^ or message encryption^10^.

Although ISO/SAE 21434^1^ was released less than one year ago, there are already research works that reviewed the standard^11^ or used it to assess the cybersecurity of automotive components like gateway ECUs^12^. The analysis in the previous work was made with the help of the ThreatGet tool, which is compliant with ISO/SAE 21434. Risk assessment methodologies based on this standard have also been discussed^13^. Automotive-specific attack surfaces were addressed and the attack feasibility was rated based on this standard^14^.

Being a topic of prime interest, a large number of IDS with various detection approaches were proposed. These approaches use various techniques to detect anomalies, like Hidden Markov Models^15^, Bloom filters^16^, frame entropy^17,18^ or the offset of ratio of responses to remote frames^19^. Some approaches also use deep learning techniques^20^, mosaic-coded convolutional neural networks^21^ or compare various machine learning algorithms^22^. Intrusion detection schemes relying on transfer learning for the Internet of Vehicles (IoV) are discussed as well^23^. Other security solutions for the in-vehicle networks include anomaly detection systems relying on Long Short-Term Memory (LSTM) autoencoders^24^ or a hybrid model based on the Support Vector Machine (SVM) and the wavelet method^25^. Other authors design intrusion detection solutions specifically for commercial vehicles buses that use the SAE J1939 standard^26^.

The use of Software-Defined Networking (SDN), federated learning and Blockchain for an IDS have been considered^27^, as well as cloud-based intrusion detection that relies on deep learning^28^. The authors of the previous work evaluated the extension of an on-board protection mechanism with a cloud-based solution to overcome the high computational demands. However, the work is focused on attacks against a robotic vehicle built with various communication interfaces but does not discuss attacks on the CAN bus as we do. Another important aspect is that the IDS can be deployed on different in-vehicle components, e.g., it can be installed on each ECU or on gateways^29^. We also discuss deployment options in our work. A comprehensive picture of the currently proposed IDS for the automobile industry is provided by recent surveys^30–32^.

Since its abrupt emergence, the Blockchain technology has started to be used not only in cryptocurrency but in many other domains as it can cover lots of specific needs for securing and distributing relevant information. In the automotive domain, the Blockchain can be used to establish the liable party (car manufacturer, driver, pedestrian, attacker, etc.) in case of accidents^33–35^.

It was also proposed for automotive manufacturing traceability systems, allowing companies to trace and document the product’s history and relevant production parameters^36^. More recently, Blockchain was also employed in a federated learning cyber-threat detection approach for intelligent transport systems, where its functionality could ensure the integrity of data and smart contracts can represent the machine learning models^37^. Other authors explore the use of Blockchain for on-demand ride-hailing platforms during the recent pandemics to increase customers’ trust by offering safe cars^38^, or develop an evolutionary game model between the car-hailing platform and the government, proposing the Blockchain as a technical governance measure^39^. Some works envision Blockchain as a foundation for developing trust management systems for the IoV^40^ or securing communication and data for unmanned aerial vehicles (UAVs)^41,42^, as well as drone node authentication in the Internet of Drone Things (IoDT)^43^. The application of the Blockchain for intrusion detection in the cyber-physical system (CPS) environment is also being studied^44^.

## CAN data collection

This section provides some background on CAN and discusses the data collection performed in three identical vehicles in order to verify that data from one of them can be used to detect intrusions on the others.

### CAN basics

**Figure 2:**
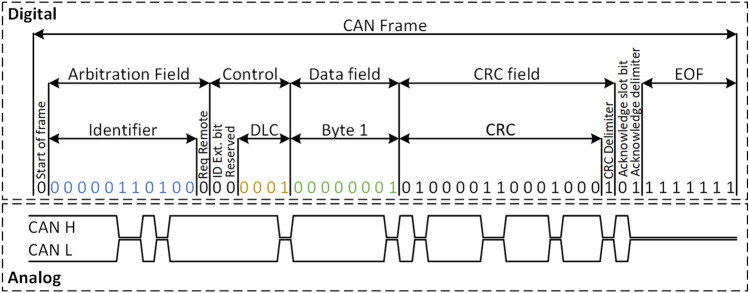
CAN 2.0 Standard Data Frame.

Despite the fact that new communication protocols were adopted in the automotive industry, e.g., FlexRay and Ethernet, the Controller Area Network (CAN), introduced by BOSCH in the 80s, remains the most commonly used in-vehicle network in modern vehicles. The CAN network, deployed as a two-wire bus that implements differential signaling, is a multi-master serial bus that enables two or more ECUs to communicate with each other. It supports bitrates up to 1 MBit/s and payloads of up to 8 bytes. The structure of the CAN data frame is presented in Fig. 2. The CAN frame is composed of an arbitration field, control field, data field and CRC field. Besides these fields, CAN frames start with a start-of-frame (SOF) dominant (logical "0") bit and end with an end-of-frame (EOF) marker that contains a sequence of 7 recessive (logical "1") bits. The CAN bus supports two message formats, i.e., a base format and an extended format. The difference between the two is the bit-length of the identifier, i.e., 11 bits in the case of base format and 29 bits in the case of extended format. One bit is used as a marker for remote transmission request (RTR), separating data frames from remote frames (which carry data requests). This bit must be recessive, a logical 1, for remote frames and dominant, a logical 0, for regular data frames. The control field of the base format frame consists of an identifier extension bit (IDE), which is always dominant in the basic format, a reserved bit and a data length field, which indicates the length of the transmitted data. The data field contains the actual payload, while the CRC (cyclic redundancy check) field contains a 15-bit CRC value and a delimiter bit. The CAN frame also contains an acknowledge slot bit (followed by a delimiter bit) used by receiving nodes to confirm the correct frame reception.

### Data collection

To support the proposal from this work, we have collected CAN data from three Dacia Duster cars (a budget family-sized SUV). Using the same car model is relevant as we need to check whether an attack on one vehicle can be identified by the IDS on other vehicles as well. Note that although the vehicles implement the same functionalities, there may be differences in the CAN bus traffic due to various manufacturing conditions which make no system perfectly identical to another. All three vehicles have similar specifications and an age difference of at most 2 years. The vehicles are equipped with 1.5 dCi diesel engines having 110 horsepower, 4 cylinders and are all-wheel drive capable. In order to distinguish between vehicles, we will reference them in this paper as Vehicle A, Vehicle B and Vehicle C.

**Figure 3:**
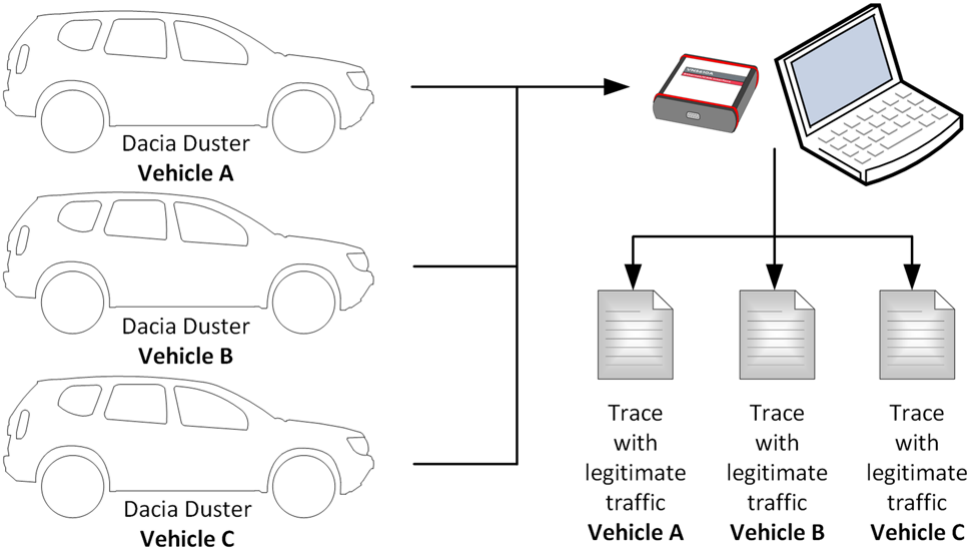
Data collection procedure.

**Figure 4:**
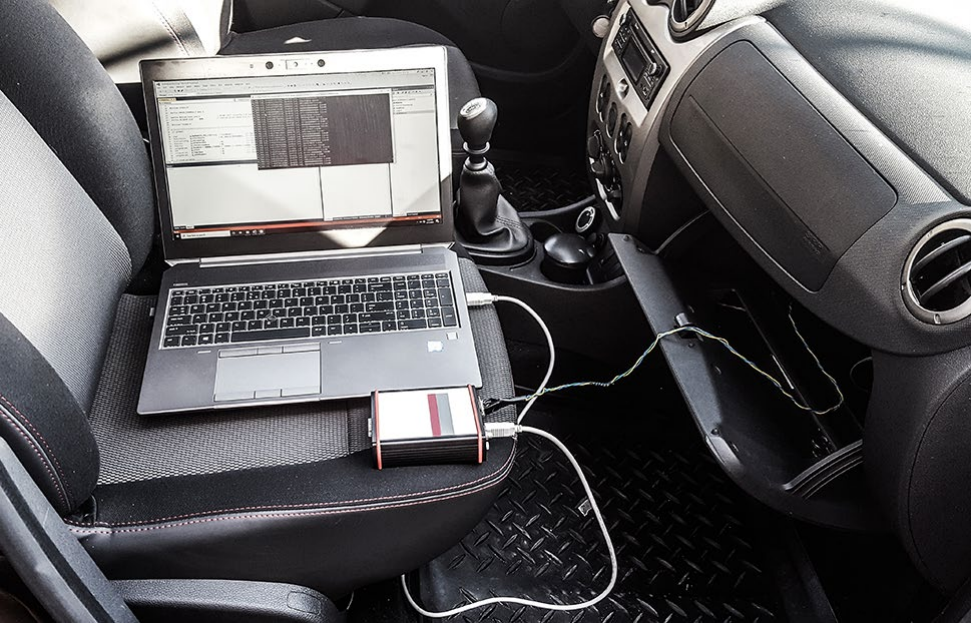
Data collection setup.

**Figure 5:**
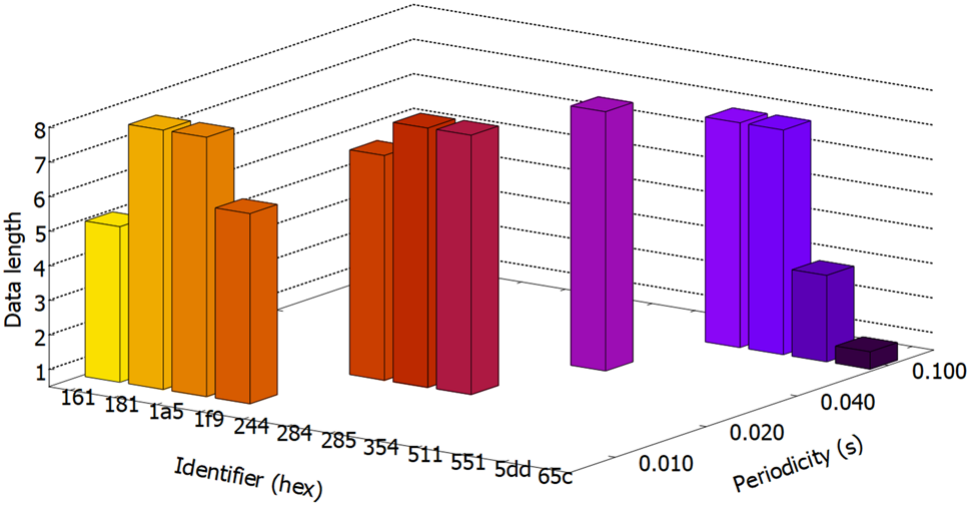
CAN frames from the three Duster vehicles.

The procedure that we used to collect legitimate CAN traffic data from the three vehicles is suggested in Fig. 3 and our setup used for data collection that consists of a laptop, a VN5610A device and a CAN cable with OBD-II connector is depicted in Fig. 4. After we collected the CAN data from all three vehicles, we performed a short comparison. Perhaps not surprising, as the three cars are identical models, we observed that the CAN logs of each vehicle contain similar CAN frames, i.e., frames with the same IDs, same lengths and having the same periodicity. There are 12 IDs in total in each log, ranging from 0 × 161 to 0 × 65C. The data length of these frames varies between 1 and 8 bytes, while their periodicity ranges from 10 to 100 ms. This is illustrated in Fig. 5 which depicts the periodicity and the data length of each CAN frame ID that is part of the collected datasets.

**Figure 6:**
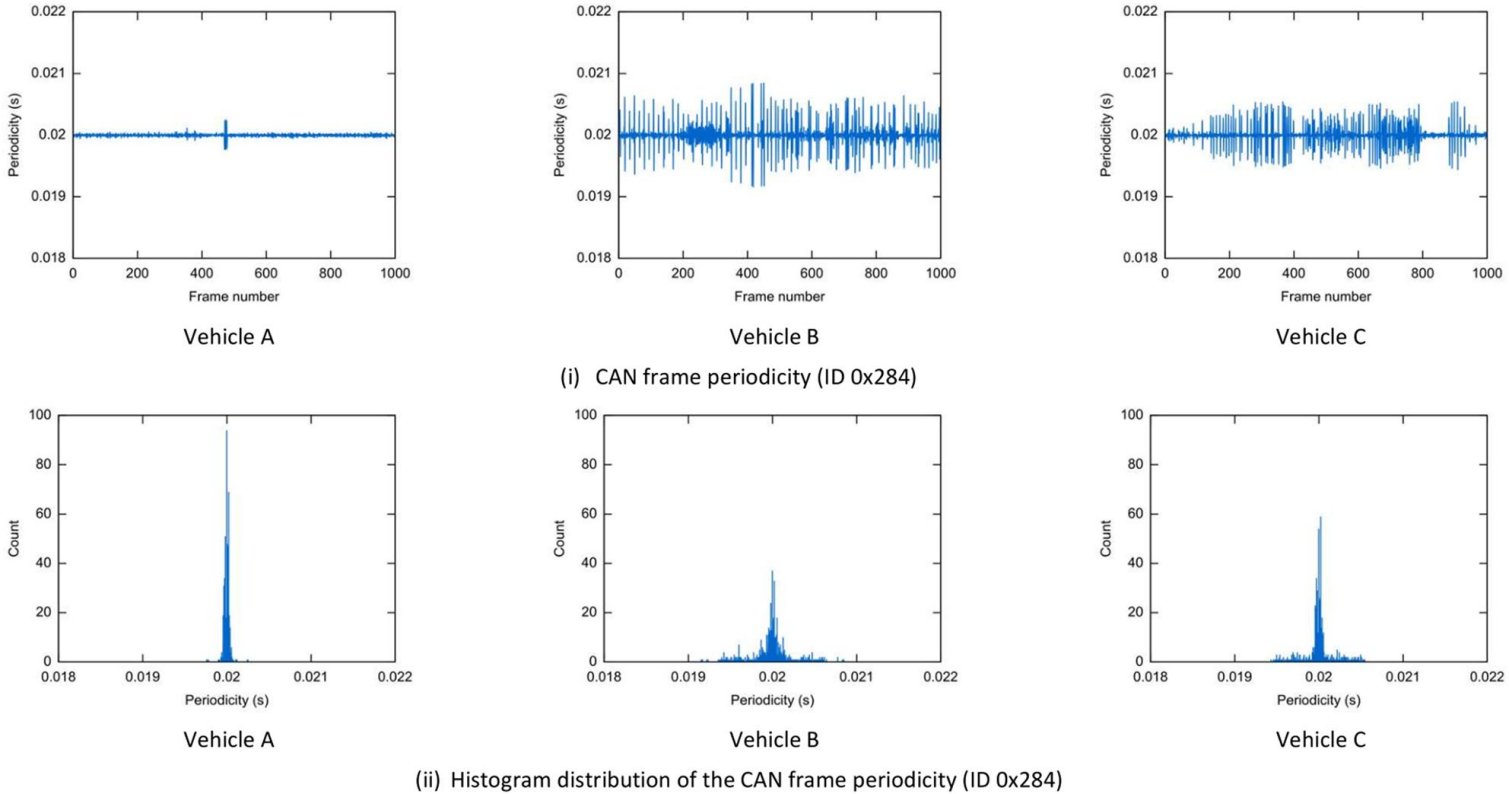
Example for the periodicity (i) and the histogram distribution of periodicity (ii) of CAN frame with ID 0 × 284 collected from vehicle A (left), vehicle B (center) and vehicle C (right).

However, on a closer inspection, even if the CAN frames are identical in terms of cycle times and IDs, traffic variations may occur between the three vehicles as we show next. Differences may be caused by various factors. For example, clock skews may cause the periodicity of CAN frames to have some deviations and driving style may also trigger distinct on-event frames that influence the arrival of the cyclic frames. We provide an example in Fig. 6 which depicts the periodicity and its histogram for the CAN frames with ID 0x284 collected in each of the three vehicles. We can observe that the periodicity has disturbances, lower in Vehicle A and higher in Vehicle B and Vehicle C.

## Adversary model and preliminary detection results

In this section, we discuss the adversary model as well as the metrics that we use to evaluate the IDS performance, then we present some preliminary detection results on traces that are augmented with attack frames.

### Adversary model

The related literature on CAN bus attacks, generally considers three types of attacks: *fuzzing*, *replay* and *flooding attacks*. Fuzzing attacks are attacks with frames containing random values in the data field and legitimate IDs. In the case of replay attacks, the adversary intercepts legitimate frames and re-sends them at a later time. In this case, the attack frames are identical with the legitimate frames thus being more difficult to detect. Flooding attacks consist of injecting frames with high priority IDs (low values), thus delaying or even stopping other ECUs from transmitting legitimate frames, eventually resulting in a Denial of Service (DoS) attack. Another kind of attack are the malfunction attacks^45^, which consist of frames with IDs chosen in advance (part of the legitimate list of IDs) and random data fields. These attacks can be regarded as a subset of the fuzzing attacks.

One specific flavor of the adversary model in this paper is that we make the adversary behavior probabilistic. There is a good practical reason that supports this choice. Since most of the CAN bus traffic is cyclic, increasing the frame rate on the bus by injecting more adversarial frames, makes the presence of an attacker more obvious. Therefore, it is expected that a more successful adversary will choose a low-rate attack to remain inconspicuous. Even more so, the attacks will be easier to detect if the adversary acts in a deterministic manner. Thus, in order to make the attacks more difficult to predict, we assume that the adversary has a fixed probability, e.g., p = 0.25, of executing an attack on a specific ID. In the experiments that follow, the attack frames are injected at random points in time between two consecutive legitimate frames carrying the same ID with some fixed probability, e.g., 25%.

Besides the attack dataset that we specifically build for this work, consisting in the aforementioned real-world CAN bus data augmented with attack frames, in order to have a common ground for comparing our IDS performance with related works, we also evaluate our IDS on the public dataset available from https://ocslab.hksecurity.net/Datasets/survival-ids. hksecurity.net/Datasets/survival-ids which were used by a prior work^45^. The data comes from three different vehicles (Hyundai YF Sonata, Kia Soul, and Chevrolet Spark) and contains three attack types: flooding, fuzzy and malfunction. Note that in this dataset fuzzy attacks refer to attacks performed with random IDs and datafields, which makes them distinct from the case of the fuzzing attacks from our adversary model (which have legitimate IDs and random datafield and are harder to detect). From this dataset, we use the malfunction attacks. We omit the fuzzy and flooding attacks from this dataset, since they are performed with IDs that do not belong to the network and are trivial to recognize with a binary search in a look-up-table containing the known IDs (there is no need for intricate machine learning algorithms).

### Metrics for IDS evaluation

In order to assess the performance of the machine learning algorithms employed in our IDS proposal, we used some of the most popular classification metrics, i.e., accuracy, precision, recall and specificity. The IDS will classify each CAN frame as a genuine or attack frame. The evaluation metrics are computed based on the following quantifiers: true positives (TP)—attack frames that were correctly classified, true negatives (TN)—genuine frames that were correctly classified, false positives (FP)—genuine frames that were incorrectly classified as intrusions and false negatives (FN)—attack frames that were incorrectly classified as genuine. Accuracy is a metric that is used to measure the percentage of frames that are correctly classified, equal to the ratio of the correctly classified frames to all processed frames: *accuracy* = (*TP* + *TN*)*/*(*TP* + *TN* + *FP* + *FN*)*.* Precision, also called positive predictive value (PPV), is the ratio of the correctly classified attack frames to all frames that were classified as attacks: *precision* = *TP/*(*TP* + *FP*)*.* Recall, also known as sensitivity, is the fraction of the reported attack frames compared to the total number of attack frames: *recall* = *TP/*(*TP* + *FN*)*.* On the opposite side, specificity is the fraction of the reported genuine frames compared to the total number of genuine frames: *specificity* = *TN/*(*TN* + *FP*)*.*

### Detection results on existing datasets and newly collected data

The framework that we propose for managing the intrusions and reporting them to the cloud, works with any detection algorithm that has been proposed in the literature. For our deployment in this work, we relied on classification trees algorithms that are supported by Matlab Statistics and Machine Learning Toolbox (https://www.mathworks.com/help/stats/classification-trees.html). The classification model was created in MATLAB and then by using MATLAB Coder we generated C code that was compiled and integrated in the Android head unit application and cloud environment to be used for intrusion classification.

**Figure 7:**
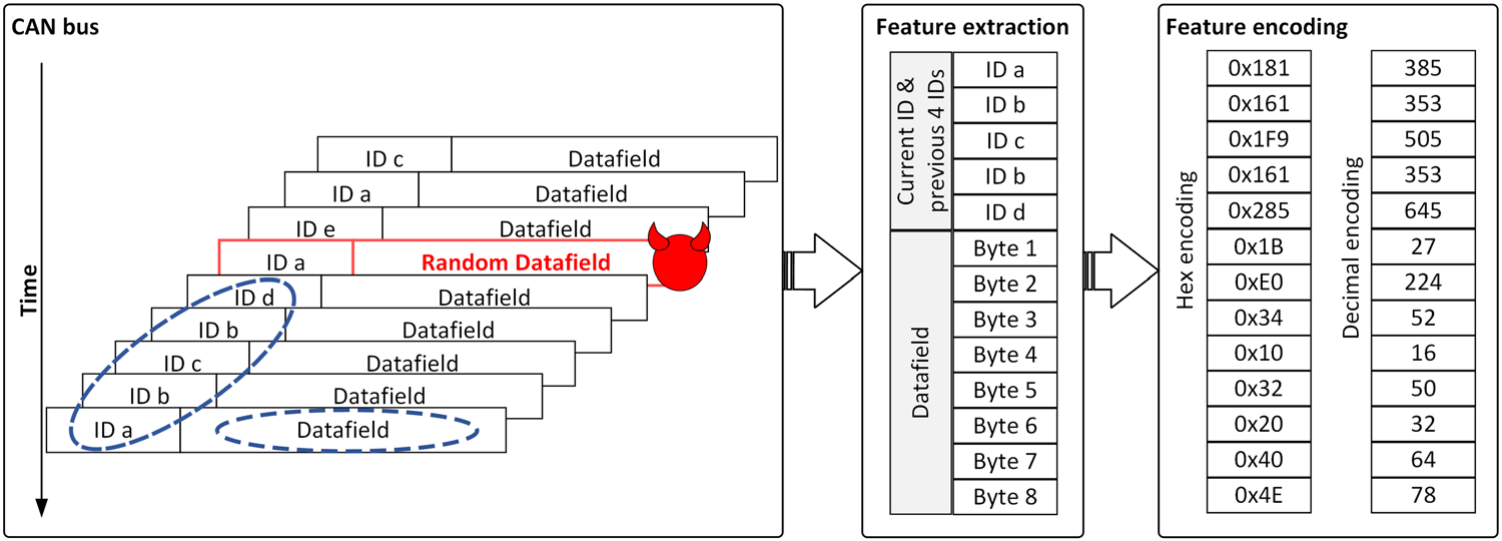
Feature extraction and encoding.

The feature extraction procedure for the IDS is graphically depicted in Fig. 7. The left side of the figure depicts a sequence of 9 CAN frames arranged in the order of their arrival. In the middle of the figure, the feature extraction is highlighted for the most recently arrived CAN frame, i.e., the frame carrying ID *a*. The classification features consist of the CAN ID of the current frame, the IDs of the previous 4 CAN frames and the datafield of the currently arrived CAN frame. For frames with a datafield length of less than 8 bytes, the features corresponding to the missing bytes are filled with zeros. The resulting dataset contains on each row the information related to the current CAN frame. All features are stored as decimal values, as suggested in the example from the right side of Fig. 7. The figure also suggests the adversary intervention by injecting a frame with random content, i.e., a fuzzing attack as discussed in the adversary model.

To serve as an initial assessment of our IDS performance and as a direct comparison, we ran the detection algorithm on the datasets with malfunction attacks made available by a previous work^45^. In this case we used 50,000 frames for training and 20,000 frames for testing. This amounts to using around 70% of the dataset for training and 30% for testing. Increasing the training dataset to 80% which is the usual amount in the related works was not necessary since the maximum score (100%) was already achieved for accuracy, precision, recall and specificity. But this mostly proves the simplicity of the attacks considered in this previous work^45^. The results are presented in the first three rows of Table 1.

**Table 1:** IDS Detection accuracy with the Classification Tree (various datasets).

Dataset	Attack type	TN	FP	FN	TP	Accuracy	Precision	Recall	Specificity
Hyundai Sonata^42^	Malfunction	16,414	0	0	3586	1.00	1.00	1.00	1.00
Kia Soul^42^	Malfunction	18,907	0	0	1093	1.00	1.00	1.00	1.00
Chevrolet Spark^42^	Malfunction	16,662	0	0	3338	1.00	1.00	1.00	1.00
Dacia Duster (our dataset)	Fuzzing	90,818	26	1	9155	0.99	0.99	0.99	0.99
Dacia Duster (our dataset)	Replay	90,540	280	1880	7300	0.97	0.96	0.79	0.99
Dacia Duster (our dataset)	Combined	88,791	1080	3011	7118	0.95	0.86	0.70	0.98

Next, we have to assess the efficiency of the IDS in terms of detection performance of fuzzing, replay and combined, i.e., replay and fuzzing, attacks. For this, we created three datasets, each of them with a different type of attack. To mount the attacks, we made use of the CANoe environment configuring two CAN nodes, the first one to replay the trace with legitimate traffic and the other one to inject attacks according to the predefined logic that we implemented in CAPL (Communication Access Programming Language). In the first dataset, we mounted fuzzing attacks with three CAN IDs, i.e., 0 × 181, 0 × 161 and 0 × 244. The same logic was applied for the second dataset, but with replay attacks. In the third dataset, we injected fuzzing attacks with CAN IDs 0 × 284 and 0 × 354 and replay attacks with CAN IDs 0 × 161 and 0 × 1A5. The detection accuracy is presented in the last three rows of Table 1. Here, we used 400,000 frames for the training phase and 100,000 frames for the testing phase (this represents an 80% cut-off for the training dataset). The fuzzing attacks are easier to be detected and therefore our IDS reached a score of 0.99 in terms of accuracy, precision, recall and specificity. In the case of replay attacks, the accuracy, precision and specificity were greater than 0.96 and the recall was 0.79. For the combined attacks, the precision was 0.86, the recall was 0.70, while the accuracy and specificity were greater than 0.95.

## System architecture and cloud-based intrusion detection

This section describes the proposed intrusion management system, addressing scalability via transfer learning and performance requirements.

### System architecture

**Figure 8:**
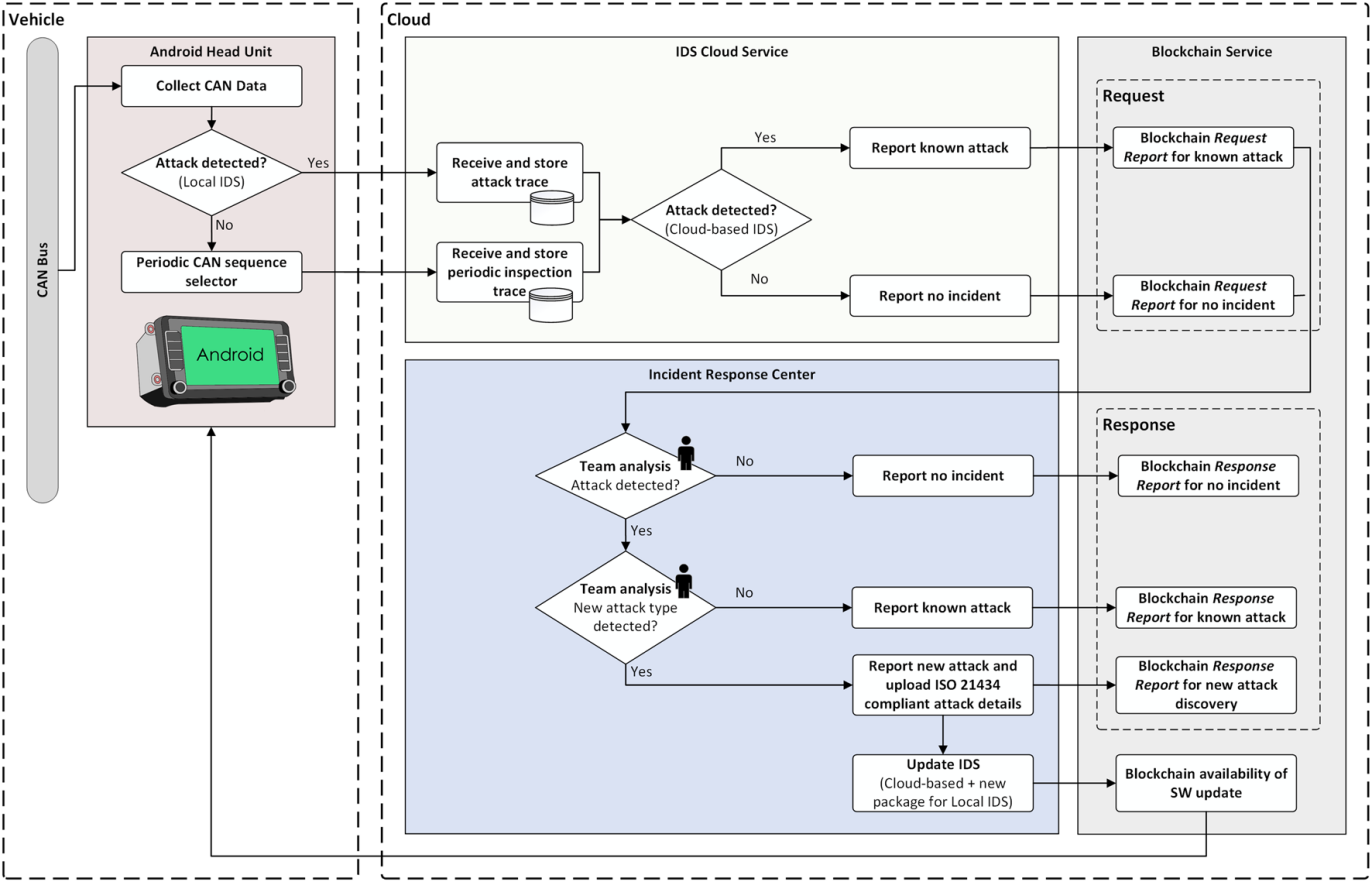
Interaction between system components.

We now provide a brief description of how each component contributes to the proposed concept. The interaction of the components is illustrated in Fig. 8.

The *Android Head Unit* monitors the traffic from the CAN bus and runs the local installed IDS. If attacks are detected, the head unit reports the attack sequences to the *IDS Cloud Service*. In addition to this, the head unit makes periodic reports with CAN data sequences which are subsequently used for sanity checks (the exact timing of such reports should be prescribed by the manufacturer, e.g., monthly, or every 5000 km, etc.). The local IDS algorithm can be updated over the air when new versions are released by the *Incident Response Center*. However, due to the prolonged life-span of a vehicle and other technical or human constraints, it may be that the head unit cannot run the most recent version of the IDS and thus cloud detection of intrusions is welcome.

The *IDS Cloud Service* receives the attack sequences, as detected by the *Android Head Unit*, and random sequences for sanity checks. These CAN sequences are stored in the cloud and evaluated against attacks using the cloud-based IDS, which is always updated to the latest version, being capable of detecting all types of attacks that were discovered so far. The output results of the cloud-based IDS, which can either indicate that a known attack was detected or that no attack is present, are documented in the form of a *Request Report* and stored on Blockchain. Each report issued by the *IDS Cloud Service* has to be further analyzed by the specialized personnel of the next actor.

The *Incident Response Center* consists of an Incident Response Team that evaluates the *Request Report* issued by the *IDS Cloud Service* and takes the appropriate incident response actions based on the impact and severity of the detected attacks. When a new report is issued by the *IDS Cloud Service*, the team checks the CAN sequences against attacks. If no attacks are detected, a *Response Report*, indicating the absence of attacks in the analyzed CAN trace, will be uploaded on Blockchain. If the presence of an attack is detected, the Incident Response Team has to assess the attack in detail, identify the impact, document the results in the *Response Report* and store it on Blockchain. Subsequently, in case of a new type of attack, the team has to update the cloud-based IDS, by retraining and testing the IDS with the new attack, and prepare the software update packet for the local IDS running on the in-vehicle head unit.

The *Blockchain service* is used to store each report, ensuring in this way that all the reported events with their corresponding information are traceable and stored in an immutable manner, providing non-repudiation from any party that is involved. In addition, this service shall also support the version control of the head unit IDS software by broadcasting notifications whenever a new release is published. However, software updates are beyond the scope of this paper and can be subject of future work.

### Scalability of the IDS over multiple vehicles

To demonstrate scalability over multiple vehicles, we performed the learning stage on Vehicle A and evaluated the IDS performance in detecting similar attacks on all three vehicles. Another important aspect regarding scalability that we must consider is the progressive learning. The IDS algorithm should be capable of learning new attacks on top of the already learned attacks. This can be done by retraining with the new attack data. But re-evaluation is necessary to check that the system does not perform worse on previously known attacks. Therefore, in our assessment, the attacks were learned progressively in three steps. As a precondition to learning of attacks, we have included in the learning phase 300,000 genuine frames from the attack-free datasets of all three vehicles so that the IDS learns the legitimate CAN traffic as there may be differences in CAN traffic between vehicles caused by various factors, e.g., clock skews, even if the vehicles are identical. Then, in the case of fuzzing attacks, we started learning with the attack that targeted CAN ID 0 × 181. As a second step, we retrain on top of the already learned attack to which we added in the learning phase a dataset containing a new attack, i.e., fuzzing CAN frames with IDs 0 × 161 and 0 × 244. In the last step, on top of these two datasets, we included another dataset that contained fuzzing attacks on the rest of the CAN frames. From each new training dataset, we used 400,000 frames for learning and we tested the IDS algorithm on 100,000 frames. The datasets are publicly available at https://github.com/andrtu2/CloudIDS to serve for future investigations.

The results are presented in Table 2. We highlighted in bold the results that come from the evaluation of the IDS algorithm on datasets from the vehicle on which the learning phase was done. The IDS algorithm proves to be highly efficient in detecting fuzzing attacks, achieving scores of at least 0.99 in accuracy, precision, recall and specificity, regardless of the assessed vehicle. For the replay attacks, we used the same approach as for the fuzzing attacks. The results are listed in Table 3, showing that the detection of replay attacks is more difficult. When assessing the vehicle that was also used for the training phase (i.e., Vehicle A), the accuracy ranges from 0.90 to 0.99, the precision from 0.87 to 0.98, the recall from 0.59 to 0.96 and the specificity from 0.97 to 0.99. The detection performance decreased by a few percents when new attacks were included in the assessment.

**Table 2:** Fuzzing attacks.

Training dataset	Testing dataset	TN	FP	FN	TP	Accuracy	Precision	Recall	Specificity
**Vehicle A—0 × 181**	**Vehicle A—0 × 181**	**96,036**	**2**	**1**	**3961**	**0.99**	**0.99**	**0.99**	**0.99**
**(+) Vehicle A—0 × 161, 0 × 244**	**Vehicle A—0 × 181, 0 × 161, 0 × 244**	**90,816**	**28**	**9**	**9147**	**0.99**	**0.99**	**0.99**	**0.99**
**(+) Vehicle A—rest of IDs**	**Vehicle A—all IDs**	**79,943**	**5**	**9**	**20,043**	**0.99**	**0.99**	**0.99**	**0.99**
Vehicle A—0 × 181	Vehicle B—0 × 181	96,088	0	3	3909	0.99	1.00	0.99	1.00
	Vehicle C—0 × 181	96,019	0	2	3979	0.99	1.00	0.99	1.00
(+) Vehicle A—0 × 161, 0 × 244	Vehicle B—0 × 181, 0 × 161, 0 × 244	90,697	1	5	9297	0.99	0.99	0.99	0.99
	Vehicle C—0 × 181, 0 × 161, 0 × 244	90,696	1	6	9297	0.99	0.99	0.99	0.99
(+) Vehicle A—rest of IDs	Vehicle B—all IDs	79,956	5	10	20,029	0.99	0.99	0.99	0.99
	Vehicle C—all IDs	80,028	9	8	19,955	0.99	0.99	0.99	0.99

Values in bold indicate that training and testing were done on the same vehicle.

**Table 3:** Replay attacks.

Training dataset	Testing dataset	TN	FP	FN	TP	Accuracy	Precision	Recall	Specificity
**Vehicle A—0 × 181**	**Vehicle A—0 × 181**	**96,107**	**64**	**118**	**3711**	**0.99**	**0.98**	**0.96**	**0.99**
**(+) Vehicle A—0 × 161, 0 × 244**	**Vehicle A—0 × 181, 0 × 161, 0 × 244**	**90,550**	**270**	**2489**	**6691**	**0.97**	**0.96**	**0.72**	**0.99**
**(+) Vehicle A—rest of IDs**	**Vehicle A—all IDs**	**78,088**	**1739**	**8083**	**12,090**	**0.90**	**0.87**	**0.59**	**0.97**
Vehicle A—0 × 181	Vehicle B—0 × 181	96,044	71	358	3527	0.99	0.98	0.90	0.99
	Vehicle C—0 × 181	95,878	87	102	3933	0.99	0.97	0.97	0.99
(+) Vehicle A—0 × 161, 0 × 244	Vehicle B—0 × 181, 0 × 161, 0 × 244	90,114	746	2982	6158	0.96	0.89	0.67	0.99
	Vehicle C—0 × 181, 0 × 161, 0 × 244	90,542	311	2811	6336	0.96	0.95	0.69	0.99
(+) Vehicle A—rest of IDs	Vehicle B—all IDs	77,608	2340	8786	11,266	0.88	0.82	0.56	0.97
	Vehicle C—all IDs	78,717	1407	9129	10,747	0.89	0.88	0.54	0.98

Values in bold indicate that training and testing were done on the same vehicle.

**Figure 9:**
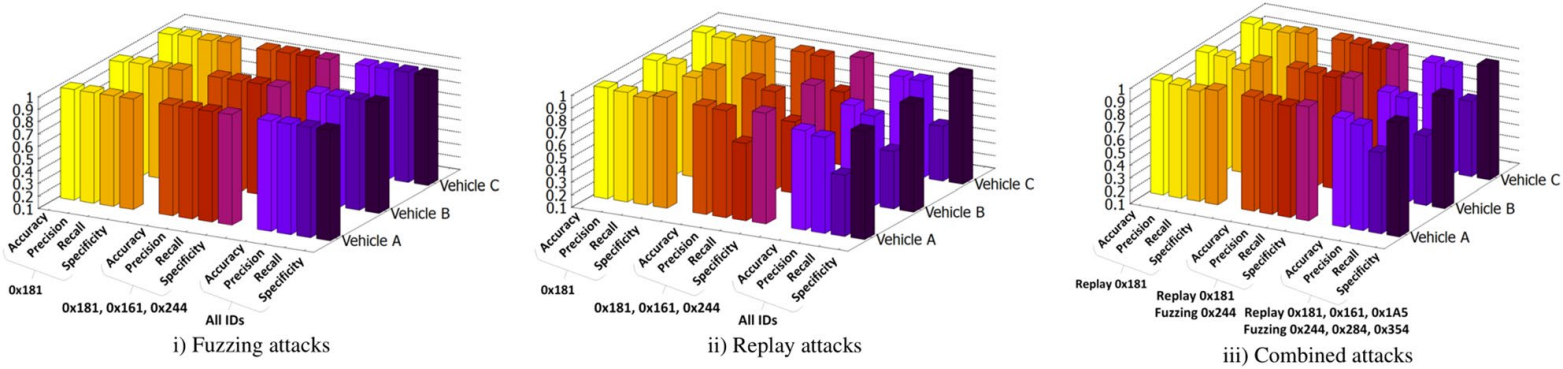
Transfer learning results: detection for attacks on all vehicles with training performed exclusively on Vehicle A.

The results from the evaluation performed on the Vehicle B and Vehicle C datasets are, in some cases, lower by a few percents than the results from Vehicle A. In the case of combined attacks, we started with learning a replay attack performed on CAN ID 0 × 181, then we added a fuzzing attack on CAN frames with ID 0 × 244, and ultimately, we added in the training phase a dataset in which two CAN IDs are attacked using replays and two CAN IDs using fuzzing. Table 4 lists the results for combined attacks. The detection performance seems to be overall better than the detection of replay attacks. The results from Vehicle A range between 0.92 and 0.99 for accuracy, precision and specificity and between 0.73 and 0.97 in the case of recall. Similar to the results from replay attacks, the detection performance drops by a few percents when assessing the datasets from Vehicle B and Vehicle C. Figure 9 depicts as barcharts the detection performance for all types of attacks, (i) Fuzzing, (ii) Replay and (iii) Combined attacks.

**Table 4:** Combined attacks.

Training dataset	Testing dataset	TN	FP	FN	TP	Accuracy	Precision	Recall	Specificity
**Vehicle A—Replay 0 × 181**	**Vehicle A—Replay 0 × 181**	**96,107**	**64**	**118**	**3711**	**0.99**	**0.98**	**0.96**	**0.99**
**(+) Vehicle A—Fuzzing 0 × 244**	**VehicleA-Replay0 × 181, Fuzzing 0 × 244**	**94,137**	**85**	**129**	**5649**	**0.99**	**0.98**	**0.97**	**0.99**
**(+) Vehicle A—Replay 0 × 161,** **0 × 1a5, Fuzzing 0 × 284, 0 × 354**	**Vehicle A—Replay 0 × 181, 0 × 161, 0 × 1a5, Fuzzing 0 × 244, 0 × 284, 0 × 354**	**84,657**	**821**	**3918**	**10,604**	**0.95**	**0.92**	**0.73**	**0.99**
Vehicle A—Replay 0 × 181	Vehicle B—Replay 0 × 181	96,044	71	358	3527	0.99	0.98	0.90	0.99
	Vehicle C—Replay 0 × 181	95,878	87	102	3933	0.99	0.97	0.97	0.99
(+) Vehicle A—Fuzzing 0 × 244	VehicleB-Replay0 × 181, Fuzzing 0 × 244	94,226	63	159	5552	0.99	0.98	0.97	0.99
	VehicleC-Replay0 × 181, Fuzzing 0 × 244	94,214	66	130	5590	0.99	0.98	0.97	0.99
(+) Vehicle A—Replay 0 × 161, 0 × 1a5, Fuzzing 0 × 284, 0 × 354	Vehicle B—Replay 0 × 181, 0 × 161, 0 × 1a5, Fuzzing 0 × 244, 0 × 284, 0 × 354	84,391	857	5282	9470	0.93	0.91	0.64	0.98
	Vehicle C—Replay 0 × 181, 0 × 161, 0 × 1a5, Fuzzing 0 × 244, 0 × 284, 0 × 354	84,653	691	4511	10,145	0.94	0.93	0.69	0.99

Values in bold indicate that training and testing were done on the same vehicle.

**Figure 10:**
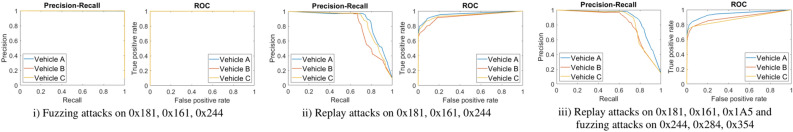
Precision-Recall and ROC curves for various fuzzing and replay attacks.

For a more in-depth interpretation of the results, we present precision-recall curves as well as ROC curves in Fig. 10, based on the datasets containing: (i) fuzzing attacks on CAN IDs 0 × 181, 0 × 161 and 0 × 244, (ii) replay attacks on IDs 0 × 181, 0 × 161 and 0 × 244 and (iii) combined attacks with replay on 0 × 181, 0 × 161, 0 × 1A5 and fuzzing on 0 × 244, 0 × 284, 0 × 354. The precision-recall curves demonstrate that a high precision can be achieved at the expense of the recall (which is due to an increase in the false negatives). This is a well-known false-positive/false-negative trade-off that can help in fine-tuning the system such that either the detection rate is high, possibly leading to more false alarms, or the detection rate is lower, but less false alarms are triggered. The ROC curves show the trade-offs between the false positives and true positives and lead to a similar interpretation as the precision-recall curves. In the case of fuzzing attacks, the curve sticks to the top left corner as the detection is almost perfect, i.e., between 99 and 100%. In the case of replay attacks, a decrease in the sensitivity is necessary in order to lower the false positives rate. This is expected since replay attacks are harder to detect as they mimic existing CAN packets.

To prevent over-fitting or selection bias, we also tried to use k-fold cross-validation (k = 5) in our Matlab models. For this, we generated five partitioned models from each original classification model, which we then evaluated using the testing datasets. Generally, the results were in the ± 4% range from the values presented in Tables 2, 3 and 4 with any of the models.

### Computational and bandwidth requirements

In order to assess the IDS computational time and bandwidth, we built an experimental model which includes a head unit and a cloud-based virtual machine. Our head unit is a PNI A8020 that runs Android 7.1 and is equipped with a Quad-core 1.63 GHz Cortex A7 CPU, 1 GB of RAM memory and 8 GB of ROM memory. For the cloud environment, we used Microsoft Azure and we have deployed a virtual machine (VM) running Windows 10. The VM is a *general purpose* VM (i.e., Standard B2ms) equipped with 2 vCPUs running at 2.80 GHz and 8 GB of RAM memory.

First, we evaluated the intrusion detection algorithm in terms of execution speed on the head unit and cloud environment. For the head unit, as we compiled the C code of the IDS algorithm into a native library and accessed it from the Android application using JNI calls, we provide two sets of results. The first set contains the IDS execution time including the JNI calls, while the second set contains only the execution time at the native layer. We assessed the execution time for the IDS algorithm trained on multiple datasets and different attacks. The results contain the average classification time per frame and are presented in Table 5. Not surprisingly, the IDS executed in the cloud performs the best, being more than ten times faster than the IDS executed on the head unit (without considering the JNI calls). The execution time of the cloud-based IDS varies between 0.62 and 171.89 µs. Based on our results, on the head unit, the JNI calls seem to bring an execution overhead between 500 and 1000 µs.

**Table 5:** IDS execution time per frame [*µs*].

Attack	Training dataset	Testing dataset	Head unit—with JNI	Head unit—C only	Cloud
Fuzzing	Vehicle A—0 × 181	Vehicle A—0 × 181	536.85	8.67	0.62
	(+) Vehicle A—0 × 161, 0 × 244	Vehicle A—0 × 181, 0 × 161, 0 × 244	538.34	19.79	1.88
	(+) Vehicle A—rest of IDs	Vehicle A—all IDs	644.97	27.24	2.34
Replay	Vehicle A—0 × 181	Vehicle A—0 × 181	657.95	105.78	9.06
	(+) Vehicle A—0 × 161, 0 × 244	Vehicle A—0 × 181, 0 × 161, 0 × 244	1661.98	942.97	58.60
	(+) Vehicle A—rest of IDs	Vehicle A—all IDs	4505.22	3699.24	171.89
Combined	Vehicle A—Replay 0 × 181	Vehicle A—Replay 0 × 181	592.79	91.37	7.81
	(+) Vehicle A—Fuzzing 0 × 244	Vehicle A—Replay 0 × 181, Fuzzing 0 × 244	748.14	116.90	9.84
	(+) Vehicle A—Replay 0 × 161, 0 × 1a5, Fuzzing 0 × 284, 0 × 354	Vehicle A—Replay 0 × 181, 0 × 161, 0 × 1a5, Fuzzing 0 × 244, 0 × 284, 0 × 354	2022.04	1126.92	72.04

Since our proposed IDS concept implies wireless connectivity between the head units and the *IDS Cloud Service*, the transmission delay and the data rate (upload speed) are important aspects that have to be discussed. Consequently, we performed some practical testing with our setup, measuring the time needed to upload data from our head unit to the Azure VM which hosts the cloud-based IDS. The measurements were done in our laboratory using 4G connectivity and the results are listed in Table 6. We measured the transmission delay by sending 1 byte of data from the head unit to the cloud application. The delay was approximately 106.56 ms. To compute the data rate, we sent data with different lengths from the head unit and measured the time required for the data to reach the cloud environment. Based on our measurements, the data rate was 1.12 Mbits/s for 100 kB of data, 2.40 Mbits/s for 1 MB of data and 2.96 Mbits/s for 10 MB of data (4G allows a maximum data rate of more than 20Mbit/s). Considering that usually the CAN bus inside vehicles is set at bit rates up to 500 KBits/s, our measurements indicate that the CAN data can be transferred to the *IDS Cloud Service* in real-time.

**Table 6:** Transmission delay and data rate (head unit to IDS Cloud Service).

Transmission delay	Data rate (100 kB blocks)	Data rate (1 MB blocks)	Data rate (10 MB blocks)
106,56 ms	1.12 Mbits/s	2.40 Mbits/s	2.96 Mbits/s

## Blockchain incident reporting

This section presents the *Blockchain Service*, which in our implementation is deployed as a smart contract on the Ethereum Blockchain^46^. We use a decentralized data storage paradigm with the goal of enforcing the resilience, availability and integrity of the attack reports.

### Blockchain reports

For each attack evaluation event performed by the *IDS Cloud Service*, an attack reporting procedure is triggered. The attack reporting procedure consists of uploading a pair formed by a *Request Report* and *Response Report* on the Blockchain. The information that has to be filled for each report is based on the guidance provided by ISO/SAE 21434^1^ which is the current standard in automotive cybersecurity.

The *Request Report* is uploaded by the *IDS Cloud Service* and contains the report unique identifier, the date when the report was made, the VIN (Vehicle Identification Number) of the vehicle from which the CAN data was extracted, the request type (which is a binary for the moment, either "No incident" if no attacks were detected or "Known attack" if the attacks were already detected by the cloud-based IDS, while new attacks are addressed later as "New attack type" by the Incident Response Team) and the hash (SHA256) of the CAN data. Subsequently, the Incident Response Team performs a detailed analysis on the CAN data considering the information from the *Request Report* and uploads the *Response Report* with the results of the analysis.

Each *Response Report* consists of the report unique identifier (that has to match the unique identifier of the corresponding *Request Report*) and report classification (which this time falls under three categories: "No incident", "Known attack" or "New type of attack"). These fields will be filled in regardless of the report classification. However, in case of attacks, a Threat Analysis and Risk Assessment (TARA) will be performed by the Incident Response Team, and subsequently the following fields (that contain details about the detected attack) will also be completed: the attack type (e.g., fuzzing, replay, combined), the asset (the frame that was attacked), the transmitter and receiver ECU(s), the security property (three properties according to ISO/SAE 21434 Confidentiality/Integrity/Availability) that is violated by the mounted attack, a detailed explanation of the possible damage scenario, the impact category and the impact rating. According to ISO/SAE 21434^1^ the impact category covers the safety (S), financial (F), operational (O) and privacy (P) aspects of assets, while the impact rating falls under the following categories: severe, major, moderate and negligible.

We will now give a synthetic example of a report that is uploaded in the Blockchain. The example consists of a situation in which the *IDS Cloud Service* reports that a known attack was detected in the received CAN trace. After the automatic inspection of the trace, the Incident Response Team confirms the attack presence and proceeds to the completion of the ISO/SAE 21434 specific fields that contain the TARA assessment. In this case, as an example, we assume that the impacted asset is the CAN frame that carries the vehicle speed signal. The receiving ECUs will use the manipulated signal values, leading to four possible damage scenarios with respect to safety or operational impact. In each of the four scenarios, the integrity of the signal is affected. The first two scenarios are related to the vehicle speed being incorrectly displayed by the instrument cluster ECU. In this situation, the driver is misinformed and could drive the vehicle faster or slower than desired. The impact rating is considered moderate for the first case, i.e., faster driving, and negligible for the opposite case, i.e., slower driving. The other two damage scenarios in which the radars receive false information are classified as severe because this could lead to the deactivation of the Driver Assistance System features or incorrect warnings displayed to the driver. The structure of a *Response Report* containing the information from the previous example is presented in Table 7.

**Table 7:** Response Report as suggested by the TARA according to ISO/SAE 21434.

Rep. no	Rep. classif	Attack type	Asset	Affected data	Transmitter	Receiver	Security property	Damage scenario	Impact category	Impact rating
1	Known attack	Fuzzing	CAN frame with ID 0 × 284	Vehicle Speed	ABS module	Instrument cluster	I	Provided speed value is less than the vehicle measured speed. Driver is misinformed and could drive the vehicle with a higher speed than the desired speed	S	Moderate
							I	Provided speed value is greater than the vehicle measured speed. Driver is misinformed and could drive the vehicle with a lower speed than the desired speed	O	Negligible
					ABS module	Radars	I	Provided speed value is less than the vehicle measured speed. Radars could deactivate the Driver Assistance System features	S	Severe
							I	Provided speed value is greater than the vehicle measured speed. Radars could provide incorrect warnings of Driver Assistance System features	S	Severe

### IDS smart contract

In order to store the previously described reports on Blockchain, we developed the *IDS Smart Contract* employing Solidity (https://soliditylang.org/) as the underlying programming language. The goal of the contract is to provide necessary API methods for uploading the report information. The following three rules are enforced by the code that we developed: (1) all uploaded data must be immutable, i.e., it cannot be subsequently altered or removed, (2) only the Incident Response Team can upload ISO/SAE 21434 assessment data and (3) only the Incident Response Team can classify reports. All data on the Blockchain is verifiable by any external party.

**Figure 11:**
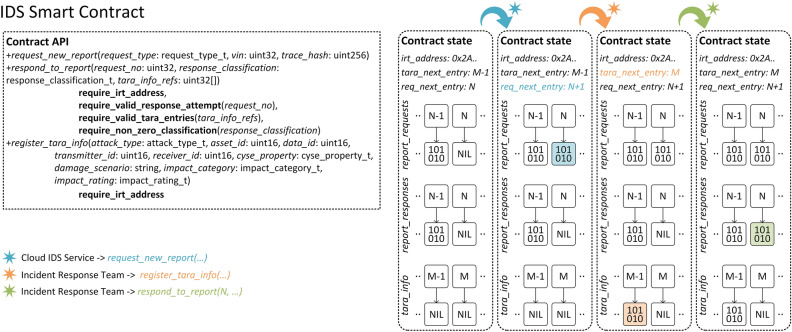
IDS Smart Contract design and state changes during method calls.

**Figure 12:**
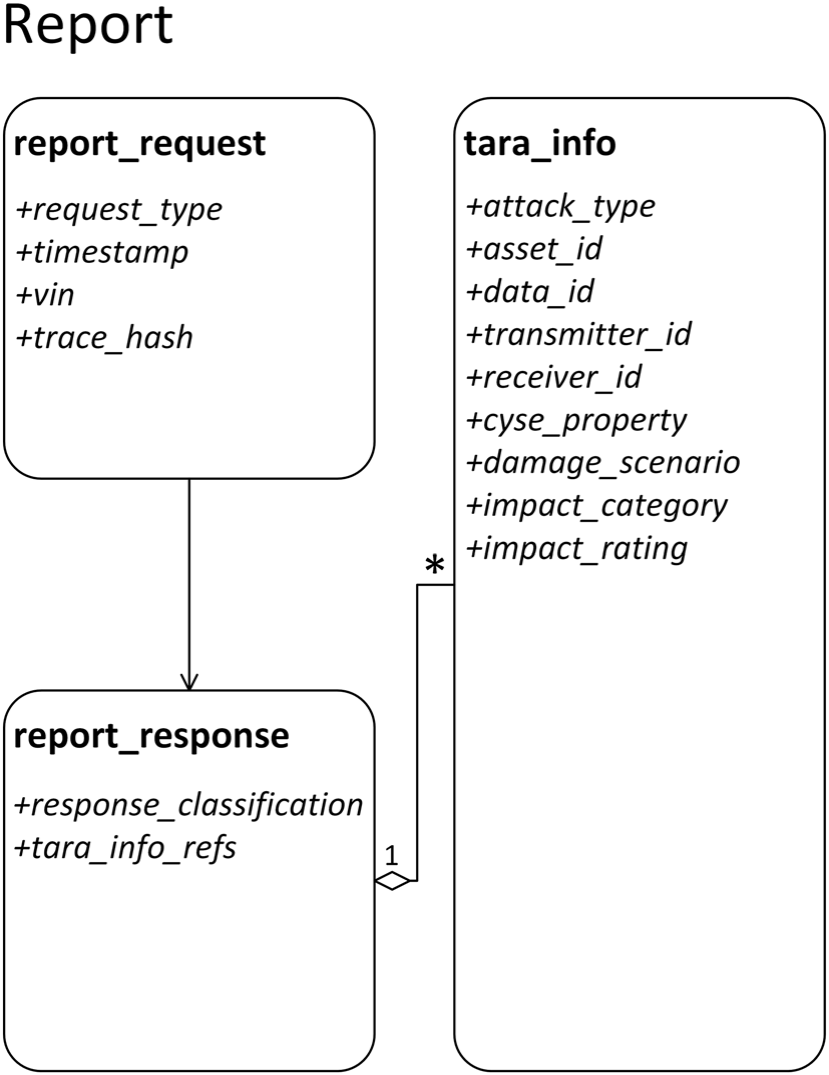
Contract composition.

Figure 11 illustrates the anatomy of the *IDS Smart Contract*. The *Contract state*, i.e., the information that gets permanently stored on the Blockchain, is composed of six public state variables. Three of them are mappings, i.e., key-value pairs, in which reports data is organized, while the others are primitives allocated for operational purposes. Listings 1 and 2 provide a comprehensive view of the state variables as well as their types. The mappings, i.e., *report_requests*, *report_responses* and *tara_info*, bind 32-bit integers to user-defined typed values. The values from the mappings are then grouped together into reports, as shown in Fig. 12.

A report is generated whenever a request is issued by the *IDS Cloud Service*, which causes a new value to be added to *report_requests*. This value incorporates the type of the request, a timestamp captured at the moment of the contract call, the VIN and the trace hash. Subsequently, the Incident Response Team classifies the event and sends a response. This leads to the addition of a new *report_response*, i.e., a value in the *report_responses* mapping, which contains the report classification and, in case of an attack, references to ISO/SAE 21434 assessment data. For every report, there is a one-to-one association between its *report_request* and *report_response* values, hence the mapping keys are sufficient in order to prevent ambiguities, e.g., for Report#4, the request and response information is mapped to key 4 in *report_requests* and *report_responses*, respectively. In contrast, an attack may affect multiple assets and cybersecurity properties, therefore, the relationship between *report_response* and *tara_info* is one-to-many. This aggregation is implemented as a table of references, i.e., *tara_info_refs*, which holds an arbitrary number of *tara_info* keys. We note that since an asset may be subject to more than one attack, referencing keys minimizes storage consumption as it prevents redundancy.

Figure 11 also exemplifies the usage of the contract API, as well as the state transitions that consequently occur. First, the *request_new_report* method is called, marking the creation of Report#*N*, i.e., a new value is added to *report_requests* at key *N*, and the update of *req_next_entry*, which always holds the number of the next report. This variable can never be decremented, thus guaranteeing the immutability of all past requests. Second, the *register_tara_info* method is called with the purpose of registering ISO/SAE 21434 assessment data. Before executing the method, the contract checks if the address of the caller matches the address stored in the *irt_address* state variable, enforcing the second rule from the beginning of this section. The value of *irt_address* is set during the execution of the contract constructor. If the verification succeeds, a new value is added to *tara_info*, mapped to key *M* − 1. Also, the *tara_next_entry* state variable is incremented so that it points to the next empty entry. This variable can never be decremented as well. Finally, the Incident Response Team responds to Report#*N* by calling the *respond_to_report* method. Here, *N* is provided as an argument. Again, the identity of the caller is checked against *irt_address* beforehand. This time however, the contract also verifies that the referenced report is not already processed, i.e., a response was added previously, and that the report classification to be stored has a non-zero value. If all checks pass, the *report_responses* variable is updated accordingly.

### Contract deployment and testing

The development, deployment and testing processes were technologically supported threefold. We began by developing the *IDS Smart Contract* in the Remix IDE environment (https://remix.ethereum.org/), an open source application that integrates cloud storage, a text editor, compilers and many other plug-ins. For deployment, Remix IDE implements compatibility with multiple cloud and local Blockchain providers.

We opted for local deployment on an Ethereum test network, created using the Ganache simulator (https://trufflesuite.com/ganache/). In addition to the server configurator, Ganache offers numerous features, including Ethereum accounts management, block explorer, mining controls and log listing. Finally, we developed Python plug-in applications that enable the interaction between the smart contract and other system components. These applications make use of the Python Web3 library (https://web3py.readthedocs.io/en/stable/) in order to read state variables and trigger transactions. We note that although we used a Windows laptop to host and benchmark the *Blockchain Service* component, a real-world deployment is not limited to this environment as the software tools that we employed are supported on all major desktop operating systems, i.e., Windows, macOS and Linux. Nevertheless, in order to benefit from an already large user base, the system integrator may choose to deploy the IDS Smart Contract on popular Ethereum networks, e.g., the Ethereum Mainnet.

**Listing 1:**
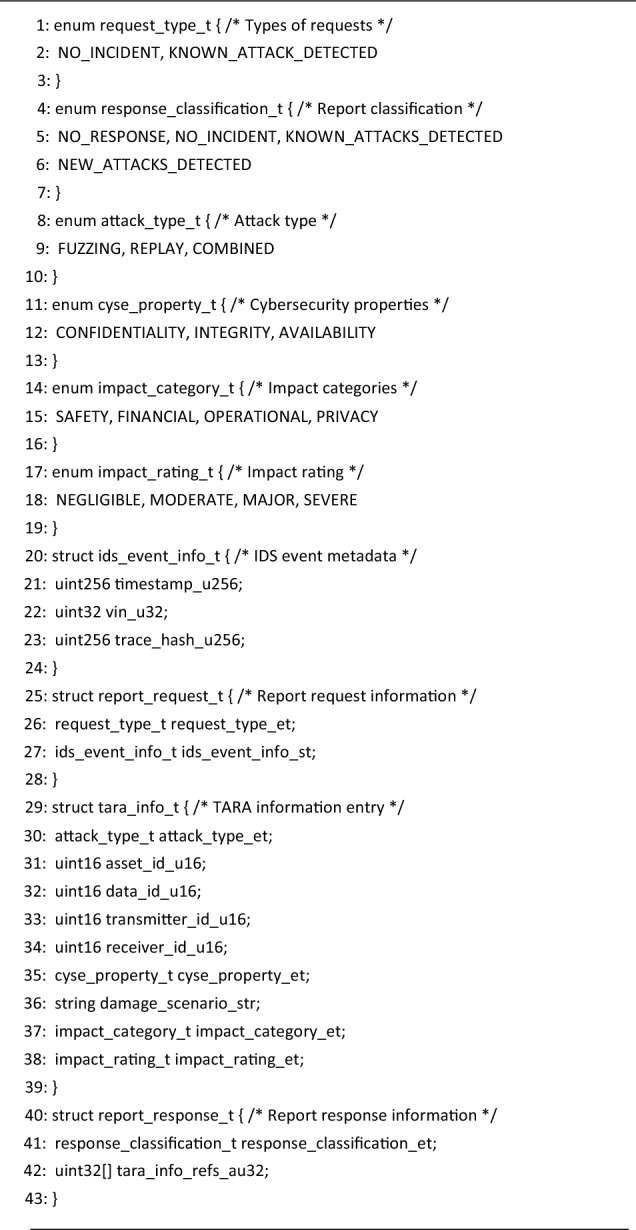
Smart Contract: defined types (Solidity).

**Listing 2:**
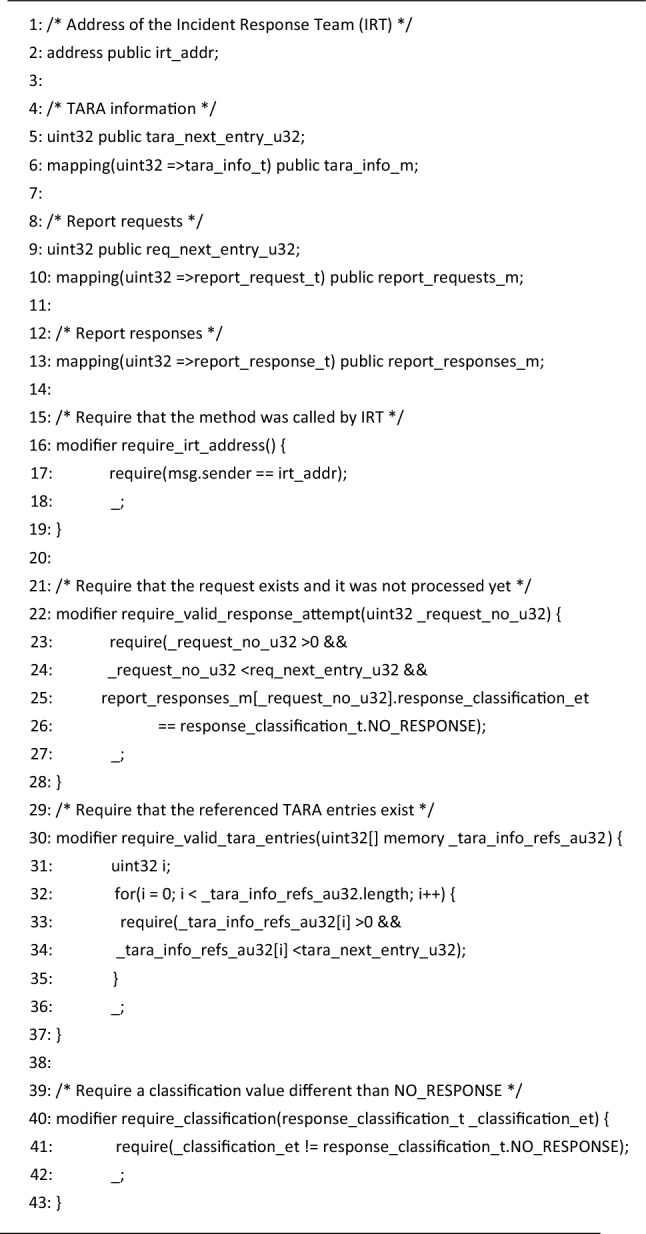
Smart Contract: variables & modifiers (Solidity).

Table 8 shows the performance of the IDS Smart Contract, measured in the average *gas consumption* per operation (in Ethereum’s terminology, *gas* represents the fee to successfully execute a contract on the Ethereum Blockchain). The first column names the evaluated operation, together with additional information regarding the uploaded data which by itself influences gas consumption, e.g., setting the state of a variable to zero consumes less gas than setting a non-zero value. The second column reports the average gas consumption as estimated by Ganache. Finally, the third column approximates the maximum supported number of transactions per second (TPS) for every operation except for the contract deployment which is required only once. At the time of writing this work, the nominal Ethereum block size is 15 million gas and the average block generation time is 13 s. These values were used for computing the TPS. Our results conclude that the operations which are expected to be performed most frequently, i.e., periodic checks labeled and classified as no incidents, are also the least expensive ones. However, the cost associated with the discovery of attacks is substantially higher, mostly proportional to the number of affected assets. Based on the tests we performed, registering a report response referencing 100 assets reaches the limit of one transaction per second.

**Table 8:** IDS Smart Contract performance.

Operation	Average gas consumption	TPS
Contract deployment	1,393,094	N/A
Register a report request, type "No incident"	92,446	12
Register a report request, type "Known attack"	111,646	10
Register a TARA entry	106,782	10
Register a report response, classified "No incident"	48,705	23
Register a report response, classified "Known attack" or "New type of attack" w. one TARA entry referenced	102,984	11
Register a report response, classified "Known attack" or "New type of attack" w. 10 TARA entries referenced	151,550	7
Register a report response, classified "Known attack" or "New type of attack" w. 100 TARA entries referenced	670,534	1

## Conclusion

Our work enriches existing in-vehicle IDS with cloud support and Blockchain reporting in accordance with the threat analysis standardized in the ISO/SAE 21434. By using the extensive computational and communication capabilities of in-vehicle head units, the support of a modern operating system, Android, the deployment of such a system becomes feasible. Our experiments prove that both the computational and communication capabilities of such units are able to support the demands of in-vehicle networks. Regarding Blockchain reporting, the gas consumption evaluated in the previous section suggests that an attack scenario with one hundred TARA entries reaches a limit of one commitment per second. Arguably, this may lead to delays for reporting attacks on larger vehicle fleets but an upper bound of one hundred entries per second for a single attack seems sufficient for current needs. If this appears as a practical limitation, multiple Blockchains may be maintained. Also, the next Ethereum generation, i.e., Ethereum 2.0, targets to dramatically increase TPS capabilities by adopting a more efficient proof-of-stake consensus layer (https://ethereum.org/en/developers/docs/consensus-mechanisms/pos/).

## Data Availability

The datasets analyzed during the current study are available from the corresponding author on reasonable request.
